# Elemental Fingerprinting of Pecorino Romano and Pecorino Sardo PDO: Characterization, Authentication and Nutritional Value

**DOI:** 10.3390/molecules29040869

**Published:** 2024-02-16

**Authors:** Andrea Mara, Marco Caredda, Margherita Addis, Francesco Sanna, Mario Deroma, Constantinos A. Georgiou, Ilaria Langasco, Maria I. Pilo, Nadia Spano, Gavino Sanna

**Affiliations:** 1 Department of Chemical, Physical, Mathematical and Natural Sciences, University of Sassari, Via Vienna 2, I-07100 Sassari, Italy; ilangasco@uniss.it (I.L.); mpilo@uniss.it (M.I.P.); nspano@uniss.it (N.S.); 2 Department of Animal Science, Agris Sardegna, S.S. 291 Sassari-Fertilia, Km. 18,600, I-07040 Sassari, Italy; mcaredda@agrisricerca.it (M.C.); maddis@agrisricerca.it (M.A.); 3 Department of Environmental Studies, Crop Protection and Production Quality Agris Sardegna, Viale Trieste 111, I-09123 Cagliari, Italy; fsanna@agrisricerca.it; 4 Department of Agriculture, University of Sassari, Viale Italia, 39A, I-07100 Sassari, Italy; mderoma@uniss.it; 5 Chemistry Laboratory, Department of Food Science and Human Nutrition, Agricultural University of Athens, 75 Iera Odos, 118 55 Athens, Greece; cag@aua.gr; 6 FoodOmics.GR Research Infrastructure, Agricultural University of Athens, 118 55 Athens, Greece

**Keywords:** pecorino cheese, elemental fingerprint, authentication, food quality, food safety, ICP-MS, ICP-OES, chemometrics

## Abstract

Sardinia, located in Italy, is a significant producer of Protected Designation of Origin (PDO) sheep cheeses. In response to the growing demand for high-quality, safe, and traceable food products, the elemental fingerprints of Pecorino Romano PDO and Pecorino Sardo PDO were determined on 200 samples of cheese using validated, inductively coupled plasma methods. The aim of this study was to collect data for food authentication studies, evaluate nutritional and safety aspects, and verify the influence of cheesemaking technology and seasonality on elemental fingerprints. According to European regulations, one 100 g serving of both cheeses provides over 30% of the recommended dietary allowance for calcium, sodium, zinc, selenium, and phosphorus, and over 15% of the recommended dietary intake for copper and magnesium. Toxic elements, such as Cd, As, Hg, and Pb, were frequently not quantified or measured at concentrations of toxicological interest. Linear discriminant analysis was used to discriminate between the two types of pecorino cheese with an accuracy of over 95%. The cheese-making process affects the elemental fingerprint, which can be used for authentication purposes. Seasonal variations in several elements have been observed and discussed.

## 1. Introduction

Dairy products and milk are among the most valuable foods due to their high nutritional value. Milk is the primary food of mammals at birth and contains, on average 4.9% of lactose, 6.2% of proteins, 7.9% of fats, 1.93 g kg^−1^ of calcium, 1.58 g kg^−1^ of phosporus and vitamins (vitamin A, 146 IU; vitamin D, 0.18 IU) [1]. Dairy products are derived from the milk of major and minor ruminant species, such as cows, buffaloes, sheep, and goats. In addition to their nutritional properties, dairy products are important for the economy and traditions of many countries. Among them, Italy is one of the most recognized in the world to produce protected designation of origin (PDO) products. The most widely renowned dairy products include Parmigiano Reggiano, Grana Padano, and Pecorino Romano.

Pecorino Romano (PR) is a sheep cheese primarily produced in Sardinia, an Italian region where the sheep dairy industry is economically relevant [2]. In fact, two other PDO sheep’s milk cheeses are produced here: Pecorino Sardo PDO (PS) and Fiore Sardo PDO. The production of PDO cheeses from sheep’s milk is the main source of income for the livestock industry on the island. Semi-extensive farming is the primary method for rearing milk sheep in Sardinia. This results in cheeses that exhibit unique sensorial properties due to the distinguishing features of pastures and climate.

Despite its relevance to the industry, the economic model is fragile due to its dependence on price fluctuations of PR. This often results in farms failing to cover production costs during times of price drops [2]. For these reasons, recent studies have aimed to enhance economic performance and sustainability of the supply chain [3], develop new marketing strategies, improve farm technologies [4], and diversify the production [5,6]. This latter strategy appears to be the most promising in reducing the reliance of milk costs on PR. Another key method for enhancing product value is to capitalize on consumer awareness of dairy quality and nutritional properties [7,8]. For instance, vitamins and minerals are of significant interest because of their association with various health benefits [7,9].

Elements such as Na, Mg, K, Ca, Fe, Cu, Zn and Se are essential for supporting the immune response, cellular processes, and antioxidant defenses [10,11]. These elements can be obtained naturally through a balanced and tailored diet, which prevents health complications caused by deficiency or excess. On the other hand, toxic elements such as As, Cd, Hg and Pb pose health risks to humans at any concentration [12]. Anthropogenic activities often lead to pollution by toxic elements, which contaminate food through water, soil, and air [13]. To ensure food safety, regulations and safety measures have been implemented to monitor and limit the presence of toxic elements in food. For instance, the European community has established maximum levels of toxic elements in food [14,15]. However, most of the toxic elements in milk and milk products are not currently regulated, such as As, Hg and Cd, while the limit for Pb in such matrices is 0.020 mg kg^−1^ [14]. Previous studies have investigated the dietary intake of toxic metals from milk and its derivatives [16,17,18].

In addition to nutritional and safety aspect, the elemental composition of foods [19] can provide valuable information for authentication [20], traceability [21], and origin assessment of dairy products [22]. The concentration of elements in foods can be affected by various factors, including climate and translocation from soil, water, and air [19]. Research has shown that elemental fingerprints of dairy products can be used to discriminate their geographical origin [23,24,25], verify their PDO authenticity [20,26,27,28,29], identify breeding methods [30], assess production processes [31], and trace the production chain [32]. Accurate sampling is necessary to achieve these goals and encompass all variables that can influence the elemental fingerprint. This includes seasonality, product processing steps, soil characteristics, pollution sources, and class variability [33].

For several decades, this research group has concentrated on the valorization [34,35,36,37,38], quality protection [39,40,41,42], classification [43], and food safety [44,45,46] of dairy products from Sardinia. Therefore, the potential of elemental analysis in food valorization and authentication was evaluated in this study by measuring the elemental fingerprint of two Sardinian PDO sheep cheeses. The concentrations of 31 elements in 200 samples of PR and PS were determined using inductively coupled plasma-optical emission spectroscopy (ICP-OES) and inductively coupled plasma-mass spectrometry (ICP-MS). The main objective was to evaluate nutritional properties and food safety to enhance products and record data for food authentication studies, e.g., for geographical discrimination. In addition, the effects of cheesemaking and seasonality on elemental fingerprints were evaluated.

## 2. Results and Discussion

### 2.1. Elemental Composition of Pecorino Romano PDO and Pecorino Sardo PDO

The elemental analysis of PR and PS was conducted using ICP-OES to determine the concentrations of macroelements (Ca, K, Mg, Na, P, and S), and ICP-MS to measure the amounts of trace elements (Zn, Fe, Mn, Cu, Se, Rb, Sr, Al, B, Co, Ni, Cr, V, Li, and Ag) and toxic elements (As, Cd, Hg, Pb, Sn, Sb, Tl, Te, Bi, and U). The results are presented in Table 1 and are expressed in kg of dry matter in the cheese.

Both cheeses had comparable levels of Ca, Mg, K and P in terms of macroelements, with an order of abundance that reflected their concentration in the original milk: Ca > P > K > S ≥ Mg [1,9]. However, due to the distinct salting process employed, the Na concentration in PR was higher than that in PS. Typically, the NaCl concentration in PR ranges from 3% to 7%, whereas in PS it rarely exceeds 2%. Even in terms of trace elements, both cheeses have a similar elemental content. Consistent with the initial milk composition, the trace elements found in highest abundance were Zn, Fe, and Cu. Both PR and PS contained similar amounts of Se, Rb, Sr, and Al. Other trace elements, such as Co, Ni, Cr, V, Li, and Ag, were present in both cheeses at levels near or below the limit of quantification.

Regarding toxic elements, both cheeses contained low levels of As, Cd and Pb. Hg was never detected. Other toxic elements, such as Sn, Sb, Te, Tl, Bi, and U, were generally either not quantified or present at very low levels. It is worth noting that the level of Te in PS was significantly higher than that in PR. However, the European Food Safety Agency (EFSA) is currently investigating the potential toxicity of Te [47].

Finally, a semi-quantitative analysis of rare earth elements (REEs) was preliminarily performed. The REEs were seldom detected above the instrumental detection limit, with a few exceptions for the LREEs. Further investigations will be conducted to optimize the limits of quantification of the analytical method and enable the determination of markers for traceability of the production chain [32].

To the best of our knowledge, the determination of trace elements in PR and/or PS has rarely been accomplished. Previous literature has mainly focused on quantifying macroelements [28,48,49], with only occasional attention given to trace elements such as Zn, Fe, Se, Cu [48], Ba [28], and Al, Ba, Cd, Co, Cr, Cu, Fe, Mn, Ni, Pb, Pt, Sr, and Zn [49]. The data obtained in this study are in good agreement with those of previous studies. Table S1 enables a comparison of the elemental compositions of PS and PR as measured in this study and in the literature.

### 2.2. Differentiation Due to Cheese-Making Process Technology

Elemental fingerprinting has been reported in the literature as a method for authenticating cheeses [23,24,26,27,28,50]. This technique is commonly used to discriminate cheeses made from milk of different animal origins [26,27,50], from different geographical areas [23,28], or from significantly different cheese-making processes, such as final moisture content and salting [24,26]. Samples were collected from various dairies in Sardinia (Italy) and varied in cheese-making technologies and production period (seasonality).

**Figure 1:**
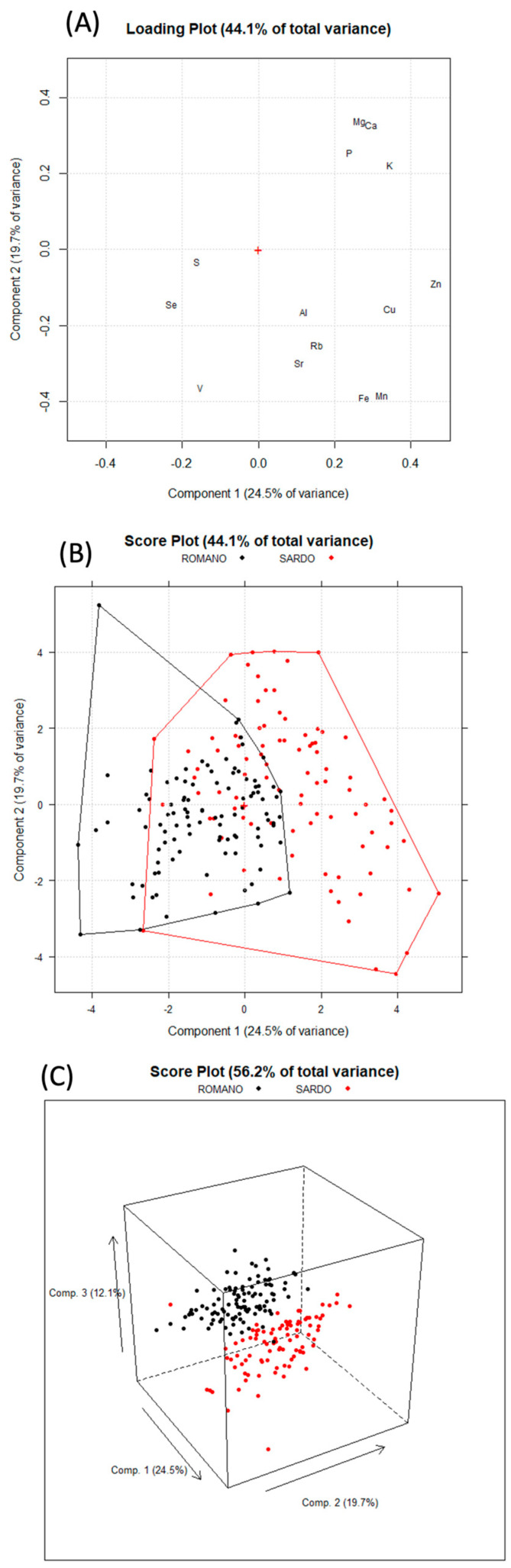
Principal component analysis performed on data obtained from the elemental determination of 14 elements on 196 pecorino samples: (**A**) loading plot; (**B**) score plot; (**C**) 3D score plot. Object colored according to cheese type.

Principal Component Analysis (PCA) was used for data visualization. The data was cleaned by removing any elements that were not quantified in at least 90% of the samples or were not significant for the analysis. Additionally, Na was excluded as a variable to eliminate the influence of the salting process. Outliers were identified and removed using T^2^ and Q statistics after performing a preliminary PCA with *p* > 0.05. The results of the PCA are presented in Figure 1.

PC1 and PC2 accounted for 24.5% and 19.7% of the total variance, respectively. Figure 1A shows that the most abundant trace elements, such as Zn and Cu, are characterized by positive values of PC1, while macroelements, including Ca, Mg, P, and K, are characterized by positive PC2 values. Looking at the score plot (Figure 1B), positive PC1 values tended to occur in the PS cluster (red samples), which was associated with a higher concentration of trace elements, while the PR cluster (black samples) tended to have negative PC1 values. The differentiation between the two clusters was accentuated upon observing PC3, which explained 12.1% of the variance (3D score plot, Figure 1C). This evidence suggests that the elemental fingerprint may be used to discriminate between the two types of cheese. Linear Discriminant Analysis (LDA) was used for classification. The MANOVA test showed a significant difference between the two groups (F (15, 180), Wilks = 0.175, approx. F = 56.78, *p* < 0.001). Prior to LDA, the dataset was randomized and split into a training set (n = 140) and a test set (n = 55). The results obtained from cross-validation and prediction are reported in Table 2.

The levels of discrimination achieved in cross-validation (97.1%) and prediction (95.7%) were highly accurate. The elemental fingerprint can discriminate between PR and PS using macro-elements (i.e., Ca, K, Mg, P, and S) and trace elements (i.e., Zn, Fe, Mn, Cu, Se, Rb, Sr, Al, Co, and V). These findings were consistent when LDA was used to analyze data from samples produced by three farms that yielded both PR and PS during the same period. The statistical significance of the data was reduced due to the smaller sample size (n = 62). However, the Principal Component Analysis (PCA) in Figure S1 showed that the samples were distinguishable, and the LDA successfully classified them with an accuracy of 98.1% in cross-validation.

Among the literature reviewed, the study by Di Donato et al. [28] was the most comparable to the present study as it adopted a similar approach for authenticating Italian Pecorino cheese. However, it is important to note that the samples in their study were geographically diverse, collected from three different regions of Italy. In contrast, the differences found in our study were solely attributed to the cheese production method used.

The study confirmed the impact of cheese-making technology on the elemental fingerprint. The differences in the chemical equilibrium of milk’s various elements, as well as the chemical form (ionic soluble or colloidal bound to milk proteins and fats), and the variations in cheese-making techniques (coagulation, wheying, and salting) could explain the distinct elemental fingerprints of the two cheeses analyzed in this research. During cheese production, the main stages that lead to a significant variance in the concentration of elements found in the cheese are salting and coagulation.

Salting triggers an osmotic phenomena, resulting in fluctuations in the levels of unbound minerals, which can cause a loss of water and cationic elements such as Na, K, Al, Cd, Co, and Rb. On the other hand, the elements bound to caseins and fats, such as Ca, P, Fe, Mg, Mn, Ni, Pt, and Zn, become concentrated.

During coagulation, there is a non-uniform distribution of elements between the curd and whey. Na and K are soluble elements and tend to be distributed in the aqueous phase (whey). On the other hand, Ca, Mg and P are associated in different proportions with the colloidal suspension of casein micelles and are more concentrated in the curd during cheese production [51]. Several studies have shown that the soluble phase of sheep’s milk may contain different percentages of Ca, Mg and P, with deviations from the total content ranging from 20–25%, 35–64% and 35–40%, respectively [52,53,54]. Currently, there is limited information available on the distribution of trace elements during sheep milk cheese production. However, data indicate that Zn and Mn are primarily distributed in the curd, accounting for about 90%, while Fe and Cu account for about 70% [52]. Fluctuations in pH, temperature, and milk storage conditions affect the equilibrium between soluble and colloidal forms. Generally, a decrease in pH and temperature shifts the balance towards soluble ionic forms, while an increase in pH and temperature favors solubilization. This results in an increase in the retention of certain elements in the curd during cheese production.

Based on these considerations, the various conditions of acidification (which are more intense in PR compared to PS), curd breaking (which is more extensive in PR compared to PS), cooking (with curd cooking at 45 °C in PR and 43 °C in PS), whey removal, heating, curd cooling, and salting may have induced differences in the balance between curd and whey. As a result, the different cheesemaking technologies favor the retention of certain elements, especially Zn, Fe, Cu, and Mn in PS compared to PR (see Figure 1). Milder acidification and breaking of the curd may have resulted in less demineralization during the production of PS. This could have led to greater retention of trace elements that are partially bound to casein micelles, such as Zn, Mg, Fe, and Cu, as previously observed. Additionally, rapid lactic fermentation, followed by effective whey removal, promotes curd demineralization [55].

### 2.3. Effect of Seasonality

In Sardinian sheep farming births are synchronized and the lactation period starts in November and ends in June–July. The chemical composition of sheep’s milk changes during this period, depending on the diet, lactation stage, and climate [4]. The concentration of minerals and major classes of compounds can be affected by the lactation stage [56]. Therefore, we evaluated the effect of seasonality on the elemental composition of PR and PS cheeses using PCA and ANOVA.

The PCA analysis in Figure S2 shows a distinct trend for Pecorino Romano PDO cheese. The loading plot indicates that PC1 describes the correlation between trace element concentrations and seasonality. Negative values indicate elements that are more abundant in PR produced in summer (V, Al, Rb, and Fe), while positive values indicate elements that are more abundant in PR produced in winter (Zn and Cu). The ANOVA results confirmed that seasonality had an impact on 12 out of 15 elements (see Figure S3). Winter-produced cheeses had the highest concentrations of Zn, Cu, K, and Mn, while spring-produced cheeses had the highest concentrations of Ca, Mg, P, and S. Summer-produced cheeses had the highest concentrations of Rb, Fe, Al, and V. The concentrations of Se, Na, and Sr were not affected by seasonal variations.

In contrast, the impact of seasonality on Pecorino Sardo PDO is relatively minor. Although the PCA did not reveal any clear trends (see Figure S4), the ANOVA indicates a significant effect of seasonality on 8 out of 15 elements (Zn, Ca, K, P, S, Cu, Rb, and V), as shown in Figure S5. Notably, the trends for these elements were like those observed for PR, with higher concentrations of Ca, P, and Mg found in PS during the spring season. Additionally, the concentration of Cu was highest in winter cheese.

As expected, both types of pecorinos exhibit similar seasonal variations in their composition, reflecting the composition of sheep’s milk. Ca is closely linked with P in casein micelles, which provide the structure and stability of the micelles. Colloidal calcium phosphate links the casein submicelles together, occupying 6% of the micellar structure. Therefore, there is a positive correlation between Ca, P, and casein content in ruminant milk [57]. Both cheeses (PS and PR) exhibit the highest concentrations of Ca, Mg, P, and S during the spring season when the amount of casein in the ewe’s milk reaches its daily maximum level [4]. S is not directly involved in the stabilization of micelles, but it is present in proteins, specifically whey proteins (cysteine and cystine amino acids). Therefore, the higher concentration of sulfur in spring cheeses could be linked to the protein concentration found in the milk of spring sheep. Despite this, the cheese still contains only small amounts of whey proteins. When examining the alkaline elements, no evident trends were found. Na cannot be evaluated due to the salting process. K remained constant in winter and spring but decreased significantly in summer. Rb in PR increased from winter to summer, but this trend was not observed in PS. This difference was likely due to different production methods. Regarding trace elements, there were two opposite trends observed. At the start of the lactation period, the concentrations of Zn, Mn, and Cu were highest. However, towards the end of the lactation period, the concentrations of Fe and V tended to increase.

The elemental composition of sheep’s milk during lactation is subject to seasonal variations, which are influenced by various factors such as the lactation stage, nutritional status of the animal, as well as environmental and genetic factors [51,58]. The mineral content in milk is weakly affected by ruminant feeding because the maternal skeleton tends to demineralize during periods when dietary mineral intake does not meet the mineral requirements of the newborn, thus, compensating for the deficit [59]. Skeletal demineralization typically occurs during periods of high mineral demand, such as early lactation and colostrum production [60]. The influence of the lactation stage on the mineral composition of milk is not well-documented. In bovine milk, Ca, P, Mg, and Na levels tend to increase towards the end of the lactation period [61]. This is likely due to increased permeability of the mammary epithelium as lactation progresses [62]. Finally, the mineral content of milk can also be influenced by the animal’s health status and genetic type. The concentration of most minerals in milk decreases when mastitis is present in the mammary gland, except for sodium and chloride ions, which increase instead [63].

### 2.4. Nutritional and Safety Aspects

**Figure 2:**
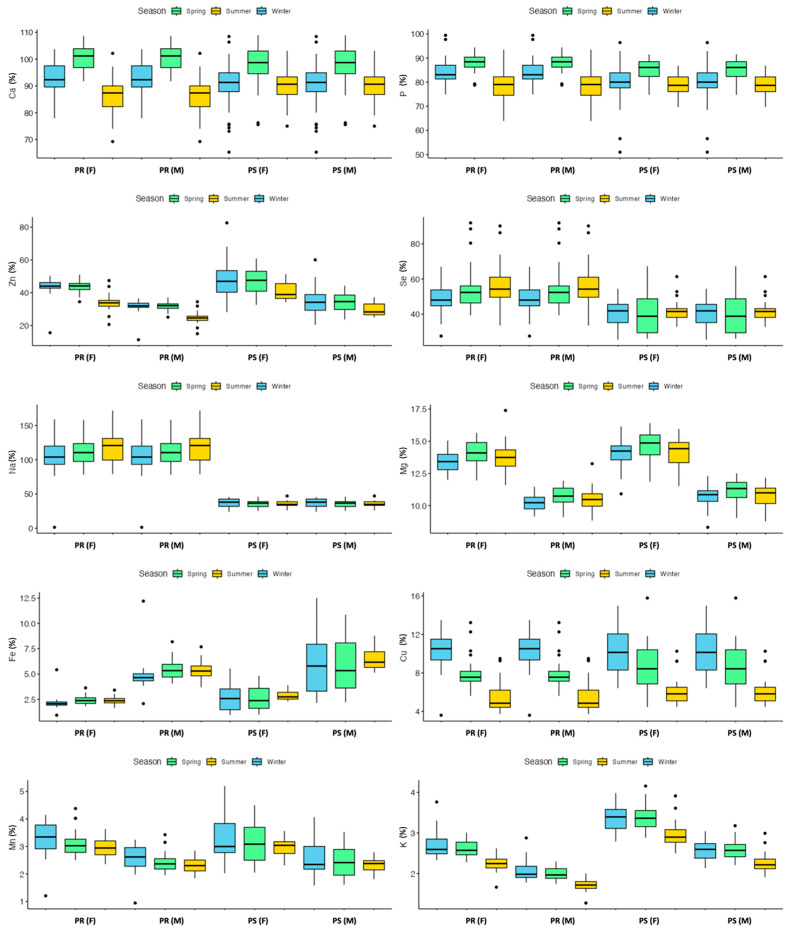
Mineral daily intakes for adult males and females (17–70 years old) for the consumption of 100 g of Pecorino Romano PDO and Pecorino Sardo PDO.

Milk and dairy products are considered highly nutritious. Mineral content is an important factor in determining food value, according to consumer preferences. Cheese is a well-known source of minerals, especially Ca, P, and Mg. Casein peptides in milk or cheese prevent the precipitation of calcium in the intestine, making it easily bioavailable [64]. Although the etiology of osteoporosis is complex, adequate calcium intake during childhood and adolescence is important for developing high peak bone mass. Maximizing bone mass early in life is considered a crucial preventive factor against osteoporosis [65]. This study’s results indicate that Pecorino Romano PDO and Pecorino Sardo PDO are sources of several nutritional elements. Figure 2 displays the daily mineral intake for both cheeses across genders. The data were calculated based on the Dietary Recommended Intakes (DRI) published by the US Department of Health and Human Services, National Institutes of Health [66].

**Figure 3:**
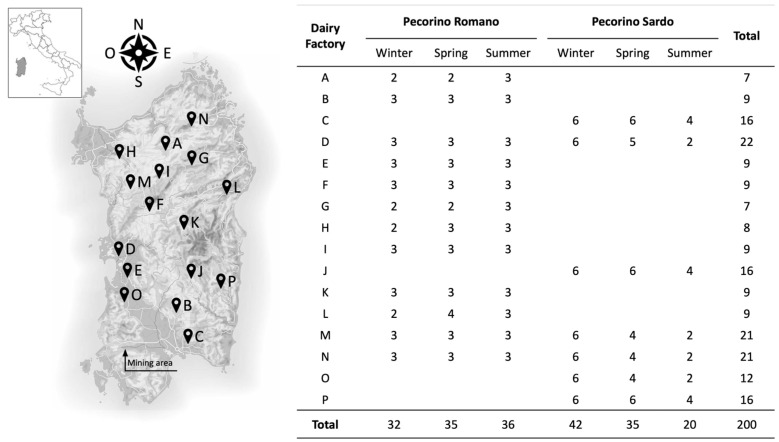
Description of pecorino sampling in terms of dairies, samples, and period of production.

Figure 2 shows that the daily consumption of both cheeses meets the Recommended Dietary Allowances (RDAs) and Adequate Intakes (AIs) for many elements (daily portion of 100 g). According to European guidelines [67,68], both cheeses are rich in Ca, P, Zn, and Se (DRI > 30%). Additionally, PR and PS cheeses are potential sources of Mg (females, DRI > 15%) and Cu (DRI > 15%). The Cu content in cheeses may vary seasonally, as shown in Figures S3–S5. Therefore, it may be possible to produce cheeses with high Cu content during the winter season. These findings are significant as they allow dairies and protection Consortia to implement nutritional labeling in accordance with European regulations [69]. As for toxic elements such as As, Cd, Hg, Pb, and Sn, both PR and PS cheeses have a high level of food safety. Additionally, the concentrations of Tl, Bi, and U were frequently below their limits of quantification. Moreover, the cheeses analyzed in this study were obtained from various locations (see Figure 3), indicating the high level of food safety in Sardinian sheep’s milk production.

## 3. Materials and Methods

### 3.1. Samples

A total of 200 samples of Pecorino cheese produced in Sardinia in 2021 were obtained from 16 dairy farms that collected milk from livestock farming in surrounding areas. Two PDO sheep cheeses were considered: PR (n = 103) and PS (n = 97). The production technologies adhere closely to the specifications outlined in their respective consortium regulations [70,71]. Pasteurized whole milk is curdled using cultures of milk enzymes from the milk’s place of origin. To produce PR, the milk is coagulated at 38–40 °C and the curd is cooked at 45–48 °C. The resulting 20–35 kg wheels mature for 5–18 months. On the other hand, PS is produced by coagulating the milk at 35–39 °C, cooking the curd at 43 °C and ripening the 1.7–4.0 kg wheels for 2–6 months. The salting process can occur through both dry and wet methods. For PR dry salting, the side and plate of the cheese receive about 2800 g of NaCl distributed thrice over a maximum span of 50 days. Alternatively, the wheels are immersed in a dynamic brine (at 12 °C and a NaCl concentration of 20–22%) for 20 days. Similarly, PS can also be salted dry or wet, but generally, it is kept in brine (24% of NaCl) for 10 h kg^−1^ of cheese at a temperature of 10–12 °C.

In this study, cheeses were produced by each dairy in three periods of the year: winter, 37%; spring, 35%; and summer, 28%. Thus, samples differed in seasoning and cheesemaking. Additionally, the cheeses varied in maturation (PR: 5–18 months, PS: 2–6 months). The wheels were first divided according to what reported in previous studies ISO 707:2008 [72]. Then, aliquots obtained were aggregated, homogenized, and stored at a temperature between −18 °C and −24 °C until analysis. Figure 3 shows the selected information on Pecorino cheese samples.

### 3.2. Instrumentation and Reagents

Elemental analysis was performed on a NexION 350X spectrometer equipped with an S10 autosampler, a glass concentric nebulizer, a glass cyclonic spray chamber, and a kinetic energy discrimination (KED) collision cell, all from Perkin Elmer (Milan, Italy). The most abundant elements were determined using an OPTIMA 7300 DV spectrometer (Perkin Elmer, Waltham, MA, USA) equipped with a GemTip Cross-Flow II nebulizer (Perkin Elmer, Waltham, MA, USA) and an autosampler (SC-2 DX, Elemental Scientific Inc., Omaha, NE, USA). To determine macroelements (Ca, K, Mg, Na, P, and S) cheese samples were previously dried in a drying oven (Memmert, Schwabach, Germany) and then calcined in a muffle furnace (Gelman Instrument, Opera, Italy). To detect trace elements (Zn, Fe, Mn, Cu, Se, Rb, Sr, Al, B, Co, Ni, Cr, V, Li, and Ag) and toxic elements (As, Cd, Hg, Pb, Sn, Sb, Tl, Te, Bi, and U) samples were digested using an ultraWAVE™ microwave single reaction chamber (SCR) system (Milestone, Sorisole, Italy) equipped with a rotor at 15 positions and 15 cm^3^ polytetrafluoroethylene (PTFE) vessels. In all procedures, type I water (resistivity > 18 MΩ cm^−1^) was produced using a MilliQ Plus System (Millipore, Milan, Italy). Hydrochloric acid (37% *w*/*w*), nitric acid (67–69% *w*/*w*, NORMATON^®^ for ultra-trace analysis), hydrogen peroxide (30% *w*/*w*, NORMATON^®^ for ultra-trace metal analysis), syringes (HENKE-JECT^®^, 20 cm^3^), syringe filters (25 mm diameter, 0.22 μm pores, nylon), paper filters (ashless, Grade 40, Whatman^®^) and metal-free falcon tubes (15 cm^3^ and 50 cm^3^) were purchased from VWR (Milan, Italy). Periodic table mix 1 (TraceCERT^®^, 33 elements, 10 mg dm^−3^ in 10% HNO_3_), periodic table mix 2 (TraceCERT^®^, 17 elements, 10 mg dm^−3^ in 5% HCl), periodic table mix 3 (TraceCERT^®^, 16 elements, 10 mg dm^−3^ in 5% HNO_3_), and certified skimmed milk powder ERM-BD151 were obtained from Sigma-Aldrich (St. Louis, MO, USA). Single standard solutions of Sc, Y, Ge, Rh, Ir, Mo, Sb, Sn, Hg, and U (100–1000 mg dm^−3^ in 2–5% HNO_3_) were obtained from LabKings (Hilversum, The Netherlands).

### 3.3. Sample Preparation

Sample preparation for macroelements analysis was performed as previously described [6]. The sample digestion for trace and toxic elements was made using an ultraWAVE™ microwave single reaction chamber (SRC) system (Milestone, Sorisole). With respect to conventional microwave instruments, SRC technology reaches higher temperatures and pressures and can manage higher amounts of samples using lower amounts of reagents [73]. In accordance with previous studies [73,74], nitric acid and hydrogen peroxide were used as oxidizing agents in this study. Approximately 0.450 g of the sample (exactly weighted on the analytical balance) was treated with 1 cm^3^ of HNO_3_ (67–69%), 2 cm^3^ of H_2_O_2_ (30%), and 4 cm^3^ of ultrapure H_2_O.

The digestion program is listed in Table 3. After cooling, the samples were collected, diluted to 15 cm^3^ using ultrapure H_2_O, and filtered using a syringe filter. The final residual acidity determined by titration with 0.1 mol dm^−3^ sodium hydroxide was 2.5 ± 0.2%. To ensure the quality of the analytical data, each digestion batch included a blank and a sample of certified reference material (CRM), ERM-BD151. The same CRM was used to assess the efficiency of microwave acid digestion in terms of matrix effect and trueness.

### 3.4. Elemental Analysis, Validation, Quality Control and Assurance

Macroelements (Ca, K, Mg, Na, P, and S) were determined using inductively coupled plasma-optical emission spectroscopy (ICP-OES), whereas trace and toxic elements were analyzed using inductively coupled plasma-mass spectrometry (ICP-MS). The instrumental parameters used for the analysis are reported in Table S2 (ICP-OES) and Table S3 (ICP-MS). Further details regarding the ICP-OES method have been previously reported [6], whereas the ICP-MS method was fully developed and validated in this study. For each PDO cheese, three samples were randomly selected and analyzed using the semi-quantitative TotalQuant^®^ method (Syngistix software v 2.3). This preliminary analysis allowed the assessment of the elements that were always within the instrumental detection limit. Excluding these, the elements of interest for a possible ICP-MS quantification were Ag, Al, As, B, Bi, Cd, Co, Cr, Cu, Fe, Hg, Li, Mn, Ni, Pb, Rb, Sb, Se, Sn, Sr, Te, Tl, U, V, and Zn. Subsequently, the possible presence of polyatomic interferences in the real matrix was ascertained, and for each element, the most suitable analysis mode (STD mode or KED mode) was determined. Validation was accomplished in terms of limits of detection and quantification, precision, and trueness. The validation parameters are listed in Table S4. The limits of detection (LoD) and quantification (LoQ) were calculated according to Currie [75]. Method repeatability (CV%_r_) was assessed by analyzing samples in triplicate within the same analytical session, whereas intermediate precision (CV%_IP_) was calculated using data obtained from different analytical sessions. Finally, trueness was evaluated by analyzing certified milk CRM ERM BD-151 and spiking tests. In the last case, for each analyte, samples were spiked three times at increasing concentration levels. The trueness measured by analyzing the CRM (Table S5) was between 89 ± 5% (P) and 114 ± 5% (Na) for the macroelements (ICP-OES method), whereas that measured for the trace and toxic elements (ICP-MS method) ranged from 92 ± 5% (Cd) to 110 ± 5% (Se). Furthermore, the recoveries measured by spiking tests ranged from 86 ± 1% (Ag) to 149± 7% (As). The recovery results (Table S4) show that the determination of more than 80% of the elements was bias-free (criteria: *t*-test, *p* = 95%). Moderate underestimation was observed for Ag and Bi, and slight overestimation for Sr and V. However, the observed bias are acceptable according to the AOAC guidelines [76]. Finally, for Na and As, a meaningful overestimation bias was observed.

Quantification was performed by external calibration using single- and multi-element standard solutions in 2.5% HNO_3_. The calibration was performed according to the expected analyte concentrations. Additionally, measurements were performed in triplicate and the data were blank-corrected. To account for signal fluctuations and matrix effects, Rh (50 µg dm^−3^) and Ir (1 µg dm^−3^) were used as internal standards. A 60-s wash with a 2% aqueous solution of HNO_3_ was introduced between consecutive samples to prevent memory effects.

### 3.5. Statistical Analysis

Data analysis was performed using R-Studio (v. 4.3.1) and Chemometric Agile Tool (CAT) [77]. The Shapiro–Wilk test was used to confirm the normal distribution of the data. ANOVA and MANOVA tests were used to compare groups, and Tukey’s HSD test was used as a post hoc test. The data set was cleaned by removing all elements that were not quantified in at least 90% of the samples or were not significant for the analysis. Elements retained for chemometric analysis were Mg, Ca, P, K, Zn, Cu, Fe, Mg, Al, Rb, Sr, S, Se, and V. Principal Component Analysis (PCA) was performed for data visualization. Linear Discriminant Analysis (LDA) was used to discriminate samples from different categories. Statistical significance was set at α < 0.05.

## 4. Conclusions

Pecorino Romano PDO and Pecorino Sardo PDO are two popular and highly regarded sheep’s cheeses. This study aimed to address the lack of knowledge regarding their elemental composition and trace element content. To achieve this, 31 elements (macro, trace, and toxic) were analyzed in a comprehensive sample of cheeses using ICP-based methods. The results showed that these cheeses are rich in essential minerals such as calcium, phosphorus, zinc, and selenium, and could potentially be a source of copper and magnesium.

Additionally, the data allowed for an assessment of the impact of cheesemaking and seasonality on the elemental composition. Linear discriminant analysis showed that the elemental fingerprint can effectively distinguish dairy products based on their production method. This result could be a useful methodology for detecting food fraud involving other cheaper pecorinos.

## Figures and Tables

**Table 1:** Elemental analysis of Pecorino Sardo PDO (n = 97) and Pecorino Romano PDO (n = 103).

Element		Pecorino Sardo PDO			Pecorino Romano PDO		
		Min	Mean ± st.dev.	Max	Min	Mean ± st.dev.	Max
Macro (mg kg^−1^)	Ca	10,000	14,000 ± 1000	16,000	10,000	14,000 ± 1000	16,000
	K	1000	1300 ± 200	1600	700	1000 ± 100	1300
	Mg	600	700 ± 50	800	500	600 ± 40	800
	Na	5000	8000 ± 1000	11,000	17,000	25,000 ± 5000	38,000
	P	8000	9000 ± 500	10,000	7000	9000 ± 700	10,000
	S	500	700 ± 100	1000	500	1000 ± 200	1400
Trace elements (μg kg^−1^)	Zn	37,000	56,000 ± 9000	78,000	18,000	47,000 ± 7500	60,000
	Fe	2600	7000 ± 3000	14,300	2400	6000 ± 950	8500
	Mn	560	850 ± 100	1250	310	800 ± 100	1150
	Cu	600	1200 ± 500	2100	500	1000 ± 350	1800
	Se	210	340 ± 90	530	220	400 ± 100	580
	Rb	1000	1700 ± 500	2600	600	1600 ± 500	2600
	Sr	7800	13,400 ± 2500	17,900	4400	14,300 ± 2500	20,500
	Al	200	6000 ± 3000	13,900	2500	6000 ± 2000	11,300
	B	<54	2000 ± 2000	7100	<54	8000 ± 8000	30,000
	Co	0.9	4 ± 1	9	1.3	4 ± 1	6
	Ni	<10	30 ± 10	60	<10	27 ± 5	40
	Cr	<3.1	40 ± 20	95	<3.1	20 ± 10	50
	V	5	10 ± 5	21	7	15 ± 5	24
	Li	<55	<55	<55	<55	<55	<55
	Ag	<1.6	5 ± 5	8	<1.6	5 ± 5	10
Toxic elements (μg kg^−1^)	As	<3.3	6 ± 1	8.4	5.7	8 ± 1	10.8
	Cd	0.5	1 ± 0.5	1.7	0.5	1.2 ± 0.5	1.9
	Hg	<30	<30	<30	<30	<30	<30
	Pb	<3.4	20 ± 10	40	<3.4	20 ± 10	45
	Sn	<2.4	20 ± 10	54	<2.4	10 ± 10	32
	Sb	<3.6	12 ± 5	16	<3.6	10 ± 5	18
	Tl	<0.5	1.9 ± 0.5	2.3	<0.5	<0.5	<0.5
	Te	<1.2	130 ± 50	220	<1.2	9 ± 5	15
	Bi	<0.5	<0.5	<0.5	<0.5	2 ± 1	2.8
	U	<0.19	1 ± 1	5.2	<0.19	2 ± 1	7.1

**Table 2:** Results of the LDA performed for the discrimination based on cheese type. Confusion matrix and accuracy in cross-validation (training) and prediction (testing).

Confusion Matrix					
Training	Romano	Sardo	Testing	Romano	Sardo
Romano	67	3	Romano	32	0
Sardo	1	69	Sardo	2	21
**Accuracy**					
Romano	Sardo	Total	Romano	Sardo	Total
95.7%	98.6%	97.1%	100%	91.3%	95.7%

**Table 3:** Cheese digestion conditions using an ultraWAVE™ SRC system (Milestone).

Step		Time (min)	Temperature (°C)
1	Heating	25	240
2	Holding	10	240
3	Cooling	ca. 30	<40

Initial pressure: 4 MPa; Release pressure rate: 0.8 MPa min^−1^; Rotor: 15 positions; Vessels: 15 cm^3^ (PTFE), Method: 0.450 g cheese + 1 cm^3^ HNO_3_ (67–69%) + 2 cm^3^ H_2_O_2_ (30%) + 4 cm^3^ H_2_O.

## Data Availability

The data presented in this study are available in article and Supplementary Materials.

## Supplementary Materials

The following supporting information can be downloaded at: https://www.mdpi.com/article/10.3390/molecules29040869/s1, Figure S1: Principal component analysis performed on Pecorino Sardo and Pecorino Romano produced by 3 farms in the same period: (a) loading plot; (b) score plot.; Figure S2. Principal component analysis performed on Pecorino Romano samples and 14 elements: (a) loading plot; (b) score plot. Object colored according to seasonality; Figure S3: ANOVA analysis of macro and trace elements in Pecorino Romano PDO as a function of the seasonality; Figure S4: Principal component analysis performed on Pecorino Sardo samples and 14 elements: (a) loading plot; (b) score plot. Object colored according to seasonality; Figure S5: ANOVA analysis of macro and trace elements in Pecorino Sardo as a function of the seasonality; Table S1: Average elemental composition of Pecorino cheeses measured in this study and from literature data; Table S2: Instrumental conditions of the ICP-OES OPTIMA 7300 DV, Perkin Elmer; Table S3: Instrumental conditions of the ICP-MS NexION 350X, Perkin Elmer; Table S4: Validation parameters of the ICP-MS method for the elemental analysis of Pecorino cheeses; Table S5: Analysis of the CRM ERM BD-151 (skimmed milk powder).
