# Supplementary material for: Elemental Fingerprinting of Pecorino Romano and Pecorino Sardo PDO: Characterization, Authentication and Nutritional Value
Source: Molecules. 2024 Feb 16;29(4):869. doi: 10.3390/molecules29040869 (PMC10892592; doi:10.3390/molecules29040869)
Supplement: Supplementary file 1 [file molecules-29-00869-s001.zip › molecules-2850406-supplementary.pdf]

Elemental fingerprinting of Pecorino Romano and Pecorino Sardo PDO: Characterisation, Authentication and Nutritional Value

Andrea Mara [1,*], Marco Caredda [2], Margherita Addis [2], Francesco Sanna [3], Mario Deroma [4], Constantinos A. Georgiou [5,6], Ilaria Langasco [1], Maria I. Pilo [1], Nadia Spano [1] and Gavino Sanna [1,*]

[1] Department of Chemical, Physical, Mathematical and Natural Sciences, University of Sassari, Via Vienna 2, I-07100 Sassari, Italy; ilangasco@uniss.it (I.L.); mpilo@uniss.it (M.I.P.); nspano@uniss.it (N.S.)
[2] Department of Animal Science, Agris Sardegna, S.S. 291 Sassari-Fertilia, Km. 18,600, I-07040 Sassari, Italy; mcaredda@agrisricerca.it (M.C.); maddis@agrisricerca.it (M.A.)
[3] Department of Environmental Studies, Crop Protection and Production Quality Agris Sardegna, Viale Trieste 111, I-09123 Cagliari, Italy; fsanna@agrisricerca.it
[4] Department of Agriculture, University of Sassari, Viale Italia, 39A, I-07100 Sassari, Italy; mderoma@uniss.it
[5] Chemistry Laboratory, Department of Food Science and Human Nutrition, Agricultural University of Athens, 75 Iera Odos, 118 55 Athens, Greece; cag@aua.gr
[6] FoodOmics.GR Research Infrastructure, Agricultural University of Athens, 118 55 Athens, Greece

* Correspondence: a.mara@studenti.uniss.it (A.M.); sanna@uniss.it (G.S.); Tel.: +39-079229500 (G.S.)

Supplementary Material

Figure S1. PCA analysis performed on Pecorino Sardo and Pecorino Romano produced by 3 farms in the same period: (a) loading plot; (b) score plot. 3

Figure S2. PCA analysis performed on Pecorino Romano samples and 14 elements: (a) loading plot; (b) score plot. Object coloured according to seasonality. 4

Figure S3. ANOVA analysis of macro and trace elements in Pecorino Romano PDO as a function of the seasonality 5

Figure S4. PCA analysis performed on Pecorino Sardo samples and 14 elements: (a) loading plot; (b) score plot. Object coloured according to seasonality. 6

Figure S5. ANOVA analysis of macro and trace elements in Pecorino Sardo as a function of the seasonality. 7

Table S1. Average elemental composition of Pecorino cheeses measured in this study and from literature data 8

Table S2. Instrumental conditions of the ICP-OES OPTIMA 7300 DV, Perkin Elmer 9

Table S3. Instrumental conditions of the ICP-MS NexION 350X, Perkin Elmer. 10

Table S4. Validation parameters of the ICP-MS method for the elemental analysis of Pecorino cheeses. 11

Table S5. Analysis of the CRM ERM BD-151 (skimmed milk powder) 12

Figure S1. Principal component analysis performed on Pecorino Sardo and Pecorino Romano produced by 3 farms in the same period: (a) loading plot; (b) score plot.

Figure S1a

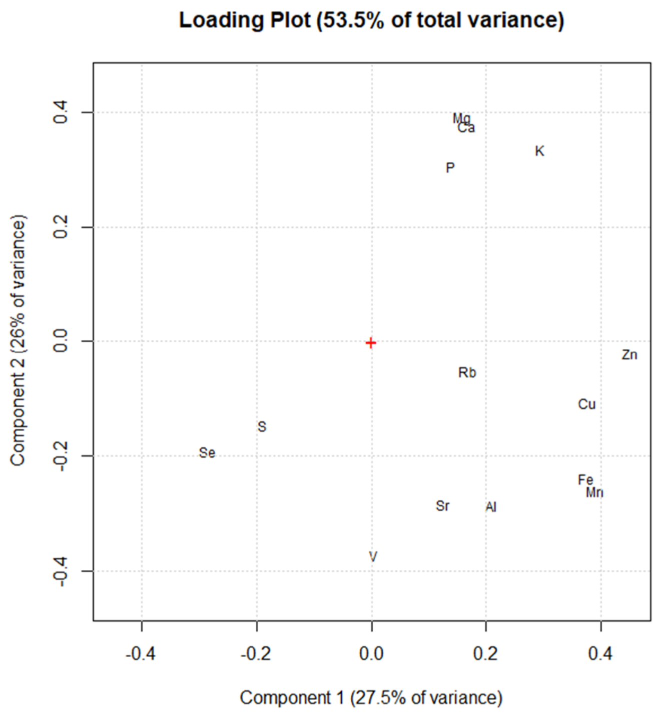

Figure S1b

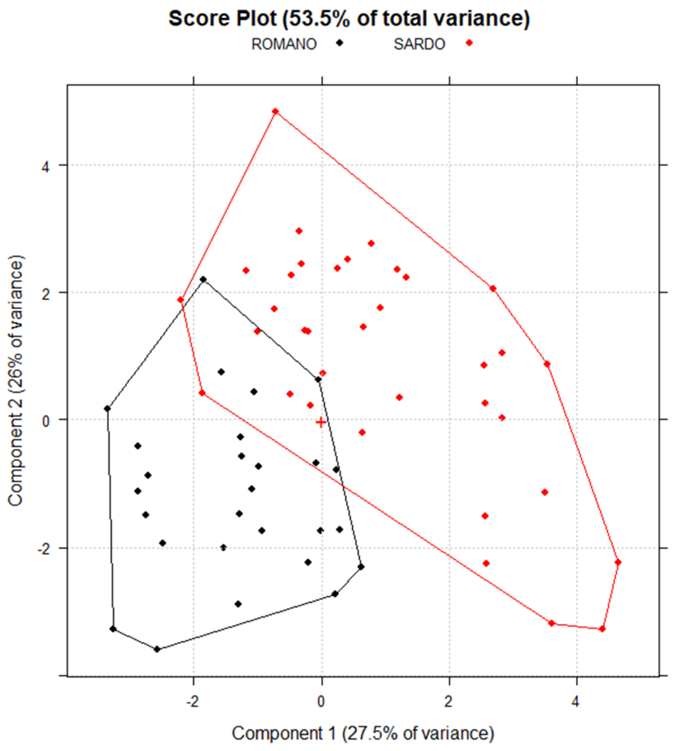

Figure S2. Principal component analysis performed on Pecorino Romano samples and 14 elements: (a) loading plot; (b) score plot. Object coloured according to seasonality.

Figure S2a

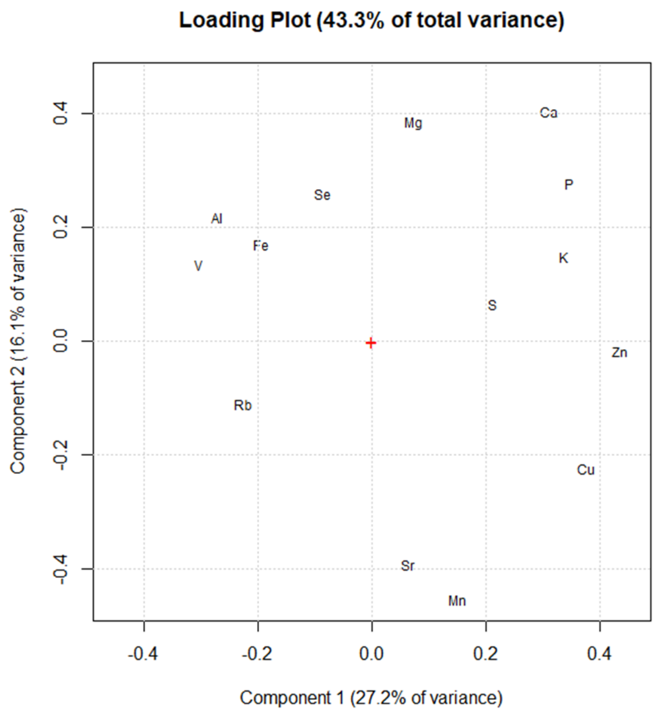

Figure S2b

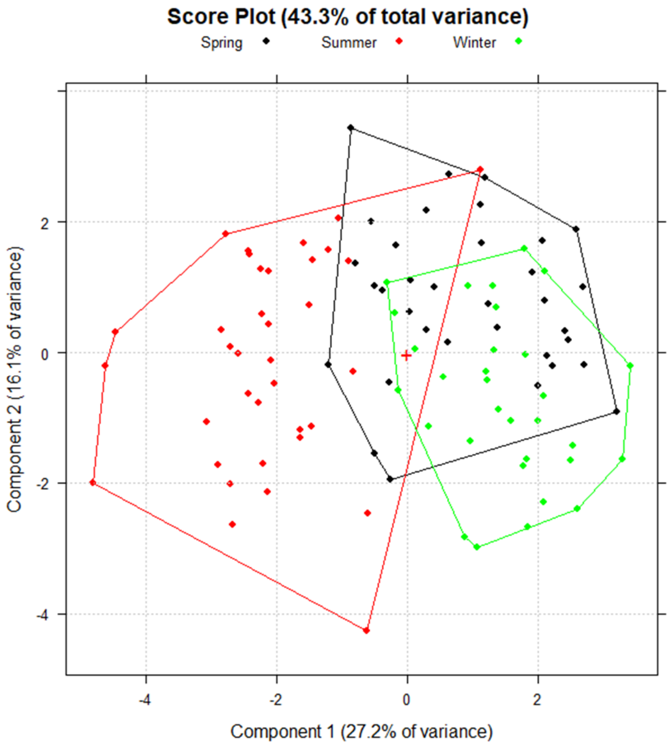

Figure S3. ANOVA analysis of macro and trace elements in Pecorino Romano PDO as a function of the seasonality.

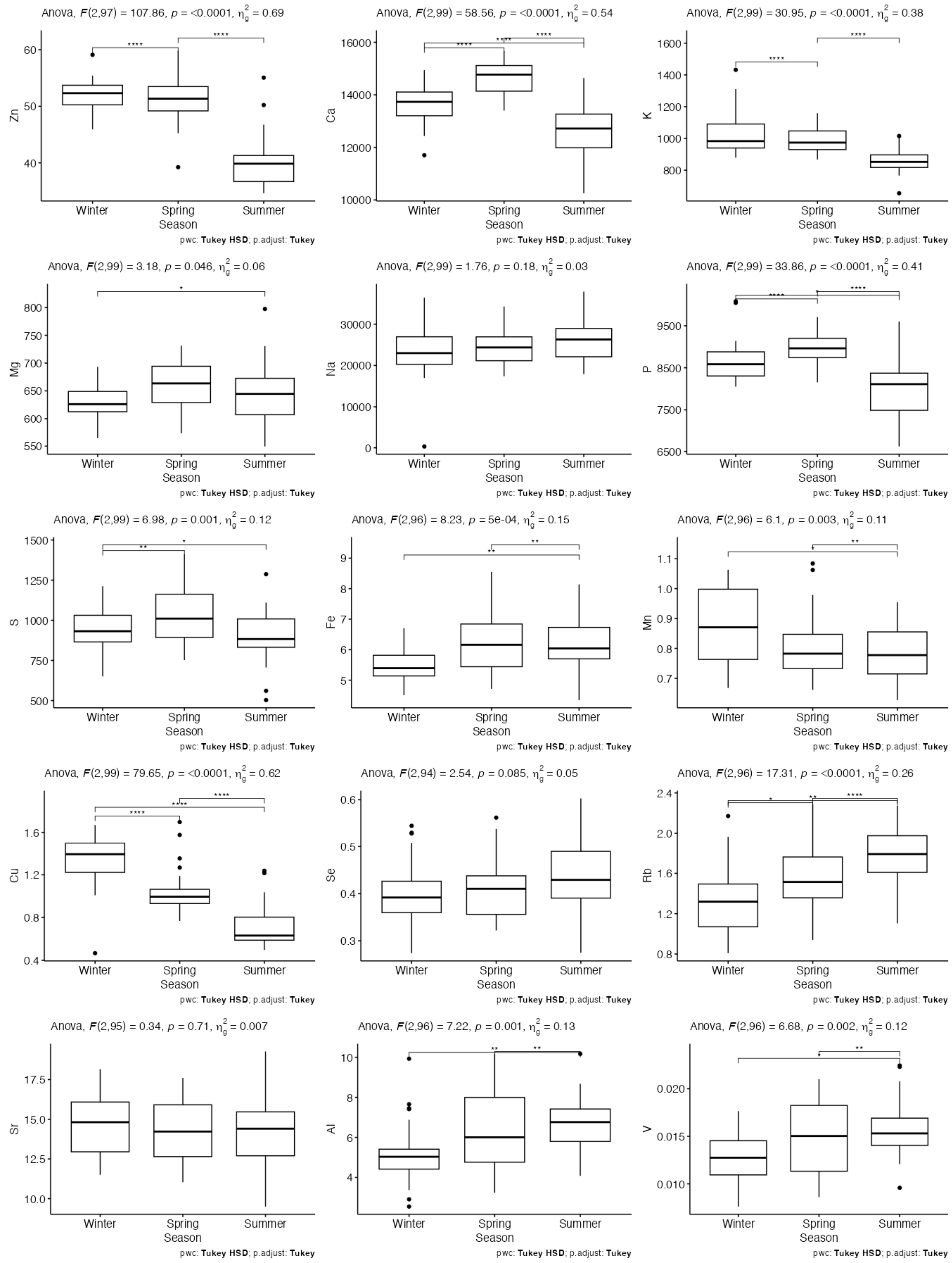

Figure S4. Principal component analysis performed on Pecorino Sardo samples and 14 elements: (a) loading plot; (b) score plot. Object coloured according to seasonality.

Figure S4a

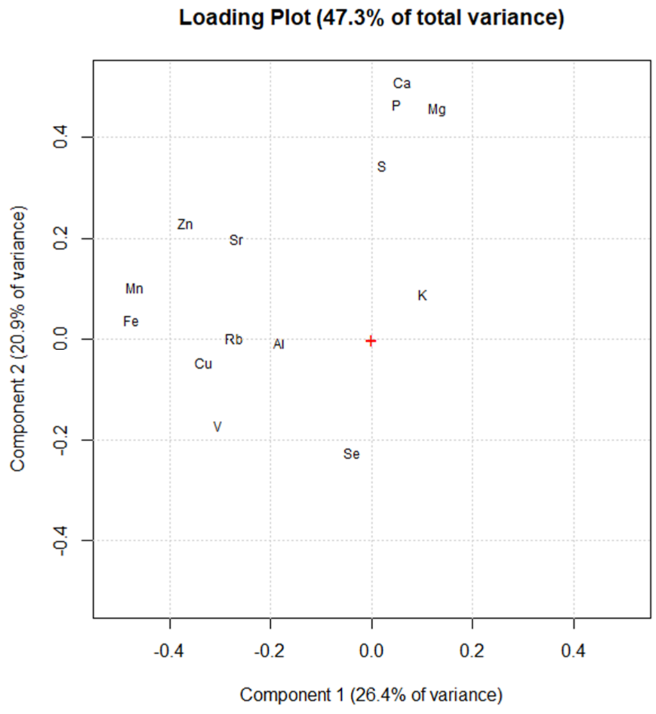

Figure S4b

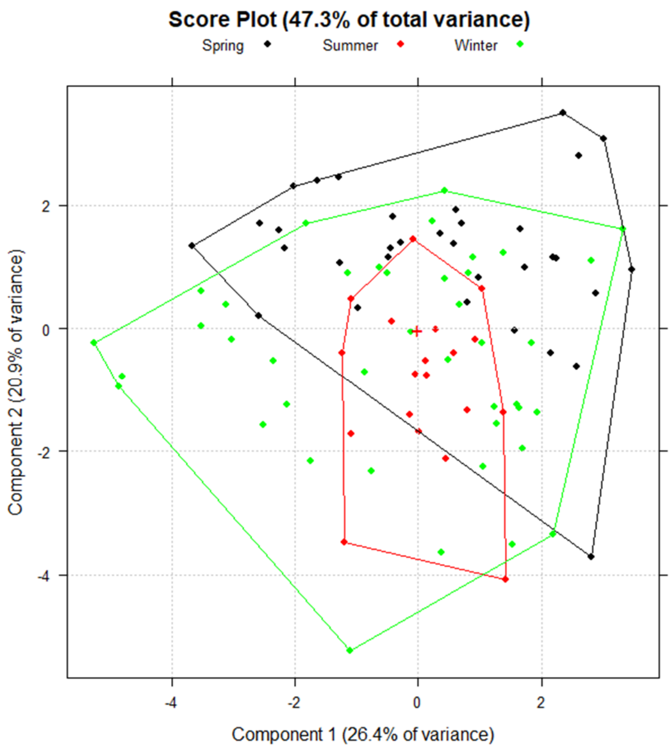

Figure S5. ANOVA analysis of macro and trace elements in Pecorino Sardo as a function of the seasonality.

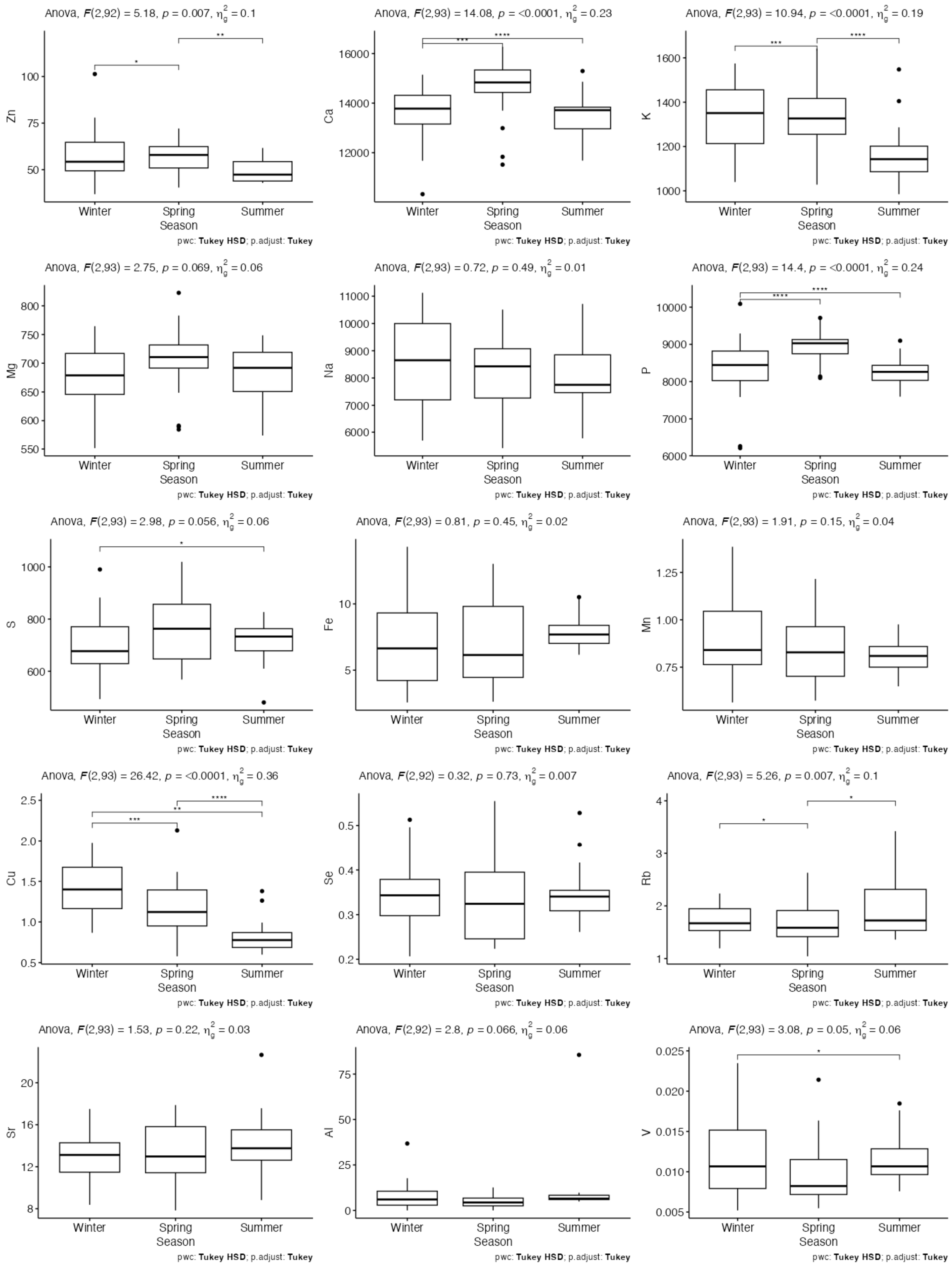

Table S1. Average elemental composition of Pecorino cheeses measured in this study and from literature data.

Elements		Pecorino Romano PDO			Pecorino Sardo PDO		Other Italian Pecorino[a]	
		Ref [57], n = 7	Ref [28], n = 17	This study, n = 103	Ref [28], n = 20	This study, n = 97	Ref [56], n = 10	Ref [28], n = 16
Macro (mg kg^{-1})	Ca		13000	14000 ± 1000	13000	14000 ± 1000	7280	12000
	K		1100	1000 ± 100	1500	1300 ± 200	1430	1700
	Mg	221	730	600 ± 40	700	700 ± 50	330	650
	Na		21000	25000 ± 5000	10000	8000 ± 1000	7820	11000
	P		9000	9000 ± 700	8000	9000 ± 500	4300	8000
	S			1000 ± 200		700 ± 100	1300	
Trace and toxic (µ g kg^{-1})	Ag			5 ± 5		5 ± 5		
	Al	2250		6000 ± 2000		6000 ± 3000		
	As			8 ± 1		6 ± 1		
	Ba	1730	3500		2700			1200
	B			8000 ± 8000		2000 ± 2000		
	Bi			2 ± 1		< 0.5		
	Cd	25		1.2 ± 0.5		1 ± 0.5		
	Co	26		4 ± 1		4 ± 1		
	Cr	40		20 ± 10		40 ± 20		
	Cu	550		1000 ± 350		1200 ± 500	600	
	Hg			< 30		< 30		
	Fe	2470	3800	6000 ± 950	2500	7000 ± 3000	3380	2400
	Li			< 55		< 55		
	Mn	270		800 ± 100		850 ± 100		
	Ni	460		27 ± 5		30 ± 10		
	Pb	19		20 ± 10		20 ± 10		
	Pt	132						
	Rb			1600 ± 500		1700 ± 500		
	Sb			10 ± 5		12 ± 5		
	Se			400 ± 100		340 ± 90	780	
	Sn			10 ± 10		20 ± 10		
	Sr	1880		14300 ± 2500		13400 ± 2500		
	Te			9 ± 5		130 ± 50		
	Tl			< 0.5		1.9 ± 0.5		
	U			2 ± 1		1 ± 1		
	V			15 ± 5		10 ± 5		
	Zn	18800	49000	47000 ± 7500	48000	56000 ± 9000	21750	40000

a) no-PDO cheeses

Table S2. Instrumental conditions of the ICP-OES OPTIMA 7300 DV, Perkin Elmer

ICP-OES OPTIMA 7300 DV, Perkin Elmer	
RF power generator (W)	1300
Ar plasma flow (dm^3 min^{-1})	15.0
Ar auxiliary flow (dm^3 min^{-1})	0.20
Ar nebulizer flow (dm^3 min^{-1})	0.80
Nebulizer	GemTip Cross-Flow II

Table S3. Instrumental conditions of the ICP-MS NexION 350X, Perkin Elmer.

ICP-MS NexION 350X, Perkin Elmer			
RF power generator (W)	1400	KED cell entrance voltage (V)	-8
Ar plasma flow ($dm^3\ min^{-1}$)	18.0	KED cell exit voltage (V)	-38
Ar auxiliary flow ($dm^3\ min^{-1}$)	1.40	Resolution (Da)	0.7
Ar nebulizer flow ($dm^3\ min^{-1}$)	0.90	Scan mode	Peak hopping
KED He flow ($cm^3\ min^{-1}$)	4.60	Detector mode	Dual
Nebulizer	Meinhardt glass	Dwell time (ms)	50
Spray chamber	Cyclonic glass	Number of points per peak	3
Skimmer and sampling cones	Nickel	Acquisition time (s)	6
Deflector voltage (V)	-10	Acquisition dead time (ns)	35
Analog stage voltage (V)	-2350	KED gas	Helium, 99.999%
Pulse stage voltage (V)	1800	Masses of optimization	^{7}Li,^{115}In and ^{208}Pb

Gas nebuliser optimisation: $^{141}Ce^{16}O^{+}/^{141}Ce^{+} < 0.03$ (NexION Setup Solution);
KED hi-flow optimisation: $^{35}Cl^{16}O^{+}/^{59}Co^{+} < 0.005$ (NexION KED Solution).

Table S4. Validation parameters of the ICP-MS method for the elemental analysis of Pecorino cheeses.

Element	Mode	Calibration Range (μg dm^{-3})	R^2	LoD (μg kg^{-1})	LoQ (μg kg^{-1})	CV%$_r$	CV%$_{IP}$	Recovery %
^{107}Ag	STD	0.1 - 50	0.99996	0.5	1.6	11%	21%	86 ± 1
^{27}Al	KED	0.5 - 200	0.99999	35	115	16%	24%	105 ± 1
^{75}As	KED	0.1 - 200	0.99996	1.0	3.3	7%	24%	149 ± 7
^{11}B	STD	0.5 - 200	0.99995	16	54	6%	13%	111 ± 4
^{209}Bi	STD	0.05 - 50	1.00000	0.1	0.5	9%	21%	88 ± 2
^{111}Cd	KED	0.05 - 100	1.00000	0.03	0.10	7%	17%	89 ± 1
^{59}Co	KED	0.1 - 200	1.00000	0.02	0.08	4%	15%	102 ± 1
^{52}Cr	KED	0.1 - 200	0.99997	0.9	3.1	9%	12%	102 ± 1
^{63}Cu	KED	0.1 - 500	1.00000	60	200	6%	16%	101 ± 2
^{57}Fe	KED	0.1 - 500	0.99996	29	90	9%	19%	112 ± 1
^{202}Hg	STD	0.1 - 50	0.99987	9	30	13%	18%	88 ± 6
^{7}Li	STD	0.1 - 200	0.99995	17	55	4%	12%	106 ± 5
^{55}Mn	KED	0.1 - 500	1.00000	0.4	1.2	5%	23%	103 ± 1
^{60}Ni	KED	0.1 - 200	0.99998	3.20	10.0	8%	17%	96 ± 2
^{208}Pb	STD	0.05 - 100	0.99996	1.0	3.4	8%	14%	95 ± 1
^{85}Rb	STD	0.1 - 500	0.99999	0.2	0.7	5%	14%	101 ± 1
^{121}Sb	KED	0.1 - 50	1.00000	1.1	3.6	8%	15%	99 ± 1
^{82}Se	KED	0.1 - 500	0.99999	2.3	7.6	9%	19%	147 ± 3
^{118}Sn	KED	0.1 - 50	1.00000	0.7	2.4	5%	14%	97 ± 2
^{88}Sr	STD	0.1 - 500	0.99999	0.8	2.6	4%	15%	109 ± 2
^{130}Te	STD	0.1 - 50	0.99999	0.4	1.2	4%	17%	107 ± 5
^{105}Tl	STD	0.05 - 50	0.99998	0.1	0.5	4%	18%	83 ± 1
^{238}U	STD	0.05 - 50	0.99998	0.06	0.19	9%	18%	101 ± 1
^{51}V	KED	0.1 - 200	0.99998	0.2	0.6	3%	18%	109 ± 2
^{66}Zn	KED	0.1 - 500	0.99997	90	300	6%	18%	115 ± 4

CV%$_r$, Variation coefficient (repeatability); CV%$_{IP}$, Variation coefficient (intermediate precision)

Table S5. Analysis of the CRM ERM BD-151 (skimmed milk powder)

Macro elements [a]	Certified value (g kg^{-1})	Experimental value (g kg^{-1}, n=3)	Trueness %
Ca	13.9 ± 0.7	14.7 ± 0.6	106 ± 4
K	17.0 ± 0.8	17.7 ± 0.5	104 ± 3
Mg	1.26 ± 0.07	1.31 ± 0.04	104 ± 3
Na	4.19 ± 0.23	4.8 ± 0.2	114 ± 5
P	11.0 ± 0.6	9.8 ± 0.6	89 ± 5
**Trace elements [b]**	**Certified value (mg kg^{-1})**	**Experimental value (mg kg^{-1}, n=3)**	**Trueness %**
Cd	0.106 ± 0.013	0.098 ± 0.005	92 ± 5
Cu	5.00 ± 0.23	4.7 ± 0.2	94 ± 4
Fe	53 ± 4	49 ± 3	92 ± 6
Mn	0.29 ± 0.03	0.28 ± 0.01	97 ± 4
Pb	0.207 ± 0.014	0.215 ± 0.005	104 ± 2
Se	0.19 ± 0.04	0.21 ± 0.01	110 ± 5
Zn	44.9 ± 2.3	44 ± 2	98 ± 5

a) ICP-OES; b) ICP-MS
